# Sexual Selection and the Evolution of Brain Size in Primates

**DOI:** 10.1371/journal.pone.0000062

**Published:** 2006-12-20

**Authors:** Michael A. Schillaci

**Affiliations:** Department of Anthropology, University of Toronto Toronto, Ontario, Canada; Wellcome Trust Sanger Institute, United Kingdom

## Abstract

Reproductive competition among males has long been considered a powerful force in the evolution of primates. The evolution of brain size and complexity in the Order Primates has been widely regarded as the hallmark of primate evolutionary history. Despite their importance to our understanding of primate evolution, the relationship between sexual selection and the evolutionary development of brain size is not well studied. The present research examines the evolutionary relationship between brain size and two components of primate sexual selection, sperm competition and male competition for mates. Results indicate that there is not a significant relationship between relative brain size and sperm competition as measured by relative testis size in primates, suggesting sperm competition has not played an important role in the evolution of brain size in the primate order. There is, however, a significant negative evolutionary relationship between relative brain size and the level of male competition for mates. The present study shows that the largest relative brain sizes among primate species are associated with monogamous mating systems, suggesting primate monogamy may require greater social acuity and abilities of deception.

## Introduction

Since Darwin's 1871 publication [1] on the evolution of humans and sexual selection, reproductive competition among males has been considered a powerful force in the evolution of primates and other mammals. The evolution of brain size and complexity in the Order Primates is widely regarded as the hallmark of primate evolutionary history. Despite their importance to understanding primate evolution, the relationship between sexual selection and brain size evolution is not well studied. With the exception of whales, primate brain evolution is unique among mammals. For primates, the evolutionary increase in brain size is often attributed to increased social complexity. Research associating increasing brain size with increasing group size and social complexity in primates predicts brain size, specifically, the size of the neocortex, will co-evolve with mating systems exhibiting social complexity [2]–[4]. In this context, larger brains are selected for because they confer greater reproductive fitness associated with increased social acuity or the ability to manipulate others within the group [2], [5]. Increases in the size of the prefrontal cortex in particular, which mediates important components of complex social behavior such as planning, working memory, memory for serial order, and language may have played an important role in human brain evolution [6].

Recent research on mating systems and brain size in a closely related mammal, bats, predicts a negative evolutionary relationship between levels of sperm competition as measured by relative testes mass, and the development of brain size stemming from an investment trade-off between two metabolically costly tissues [7]. The results from that study indicated that while species with mating systems that include multiple copulations by males has no evolutionary impact on relative brain size, mating systems with multiple matings by females do influence brain size evolution. Bat species with mating systems based on female promiscuity were associated with smaller brains and larger testes, while species with mating systems based on female fidelity were associated with significantly larger brains and smaller testicles [7].

The present research investigates the evolutionary relationship between brain size and two components of primate sexual selection in primates 1) sperm competition as measured by relative testes size, and 2) male competition for mates estimated from the level of sexual mass dimorphism.

## Results

Results from the analyses of covariance (ANCOVA) estimating the relationships of female promiscuity and mating system with brain size and testes size indicated mating system was associated significantly with brain size in primates after accounting for body mass, while female promiscuity was not (Table 1). After accounting for body mass, both mating system and female promiscuity were associated significantly with the level of sperm competition estimated from testes size. Although male body mass was associated with male competition for mates as measured by mass dimorphism, mating system and female promiscuity were not. This pattern of association remained unchanged when humans are excluded from the analysis.

**Table 1:**
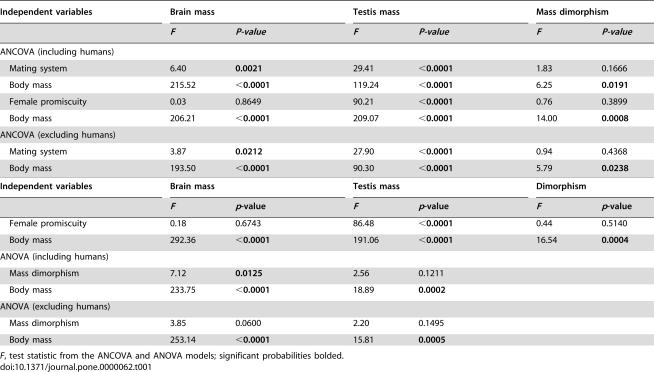
Results of analyses of covariance (ANCOVA) and variance (ANOVA) for the dependent log_e_-transformed variables: brain mass, testis mass, and mass dimorphism on measures of mating system and female promiscuity with body mass treated as a covariate.

Independent variables	Brain mass		Testis mass		Mass dimorphism	
	*F*	*P-value*	*F*	*P-value*	*F*	*P-value*
ANCOVA (including humans)						
Mating system	6.40	**0.0021**	29.41	**<0.0001**	1.83	0.1666
Body mass	215.52	**<0.0001**	119.24	**<0.0001**	6.25	**0.0191**
Female promiscuity	0.03	0.8649	90.21	**<0.0001**	0.76	0.3899
Body mass	206.21	**<0.0001**	209.07	**<0.0001**	14.00	**0.0008**
ANCOVA (excluding humans)						
Mating system	3.87	**0.0212**	27.90	**<0.0001**	0.94	0.4368
Body mass	193.50	**<0.0001**	90.30	**<0.0001**	5.79	**0.0238**

**Figure 1:**
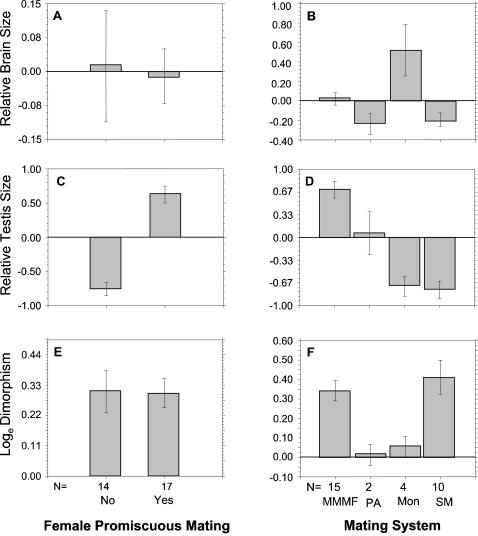
Error-bar plots of residuals from the least-squares regressions of brain (a,b) and testis (c,d) weights on body weight, and sexual mass dimorphism values (e, f) by mating system and female promiscuity determinations. Variables were log_e_–transformed prior to regression analysis. Error bars represent one standard error of the mean. Mating system: MMMF, multi-male/multi-female; PA, polyandrous; Mon, monogamous; SM single male.

Results from an analysis of variance (ANOVA) derived from multiple regression models with mass dimorphism and male body mass as independent variables, and brain size and testis size as dependent variables, indicated that the level of male competition for mates had a significant association with brain size but not with testis size. When humans are excluded from the analysis, however, the effect of male competition on relative brain size is not significant (*p* = 0.060). The regression coefficient (β) from the multiple regression model indicated an inverse relationship between relative brain size and mass dimorphism when humans are included (whole model adjusted *r* ^2^ = 0.904, β = −0.7693, *p* = 0.013), and excluded (whole model adjusted *r* ^2^ = 0.923, β = −0.4841, *p* = 0.060), from the analysis. The ANCOVA results estimating the influence of mating system and female promiscuity on primate brain size and testis size were supported by informal visual comparisons of residuals from least-squares regressions of testis and brain weights on body mass (Fig. 1). These residuals represented a measure of testis and brain size relative to body mass. A formal comparison across mating systems indicated that there are significant differences in sexual dimorphism and in regression residuals describing relative brain size and relative testis size (Table 2). A comparison between the two levels of promiscuity indicated that there were not significant differences in relative brain size and levels of dimorphism. Predictably, there was a difference between the two levels of promiscuity in relative testis size. A reanalysis of residuals from regression models excluding humans yielded similar results.

**Table 2:**
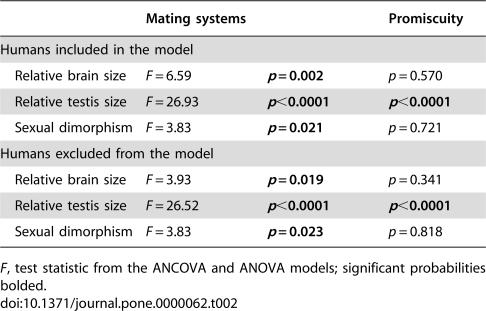
Results of the analysis of variance (ANOVA) comparing relative brain size, relative testis size, and sexual dimorphism across mating systems, and Mann-Whitney comparisons between promiscuity levels.

	Mating systems		Promiscuity
Humans included in the model			
Relative brain size	*F* = 6.59	***p*** ** = 0.002**	*p* = 0.570
Relative testis size	*F* = 26.93	***p*** **<0.0001**	***p*** **<0.0001**
Sexual dimorphism	*F* = 3.83	***p*** ** = 0.021**	*p* = 0.721
Humans excluded from the model			
Relative brain size	*F* = 3.93	***p*** ** = 0.019**	*p* = 0.341
Relative testis size	*F* = 26.52	***p*** **<0.0001**	***p*** **<0.0001**
Sexual dimorphism	*F* = 3.83	***p*** ** = 0.023**	*p* = 0.818

*F*, test statistic from the ANCOVA and ANOVA models; significant probabilities bolded.

**Figure 2:**
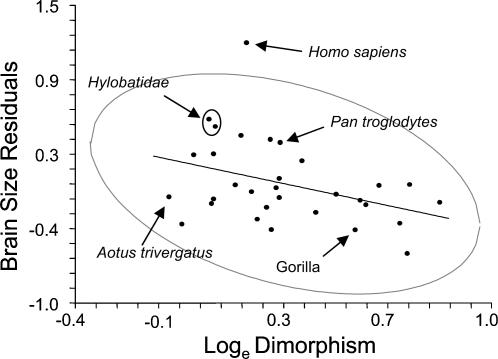
Bivariate plot of relative brain size regressed on dimorphism. Gray oval represents the 95% confidence ellipse of the bivariate distribution. The positions of all monogamous genera (*Homo, Hylobates, Aotus*), chimpanzees and gorillas are labeled for reference.

Relative brain size exhibited a significant negative Pearson correlation coefficient with mass dimorphism but not with relative testis size (Table 3). Not surprisingly, body mass exhibited a significant correlation with mass dimorphism. After controlling for the effects of shared evolutionary history, or phylogenetic inertia, independent contrasts for relative brain size exhibited a significant negative correlation with mass dimorphism contrasts, but no correlation with relative testis size contrasts. The bootstrap regression of relative brain size contrasts on mass dimorphism contrasts yielded a negative slope value (*b*) significantly less than zero (*b* = −0.5849, *r* = −0.396, *p* = 0.026). This slope value generated from the independent contrasts was not significantly different from the estimate calculated using data not corrected for phylogenetic effects (*b* = −0.5360, 95%CI−0.9189, −0.0565, *r* = −0.378, *p* = 0.039) (Fig. 2). The regression of relative testis size contrasts on mass dimorphism contrasts yielded a non-significant slope value (*b* = −0.052, *r* = −0.020, *p* = 0.881), as did the regression of relative brain size contrasts on contrasts for relative testis size (*b* = 0.0074, *r* = 0.014, *p* = 0.856).

**Table 3:**
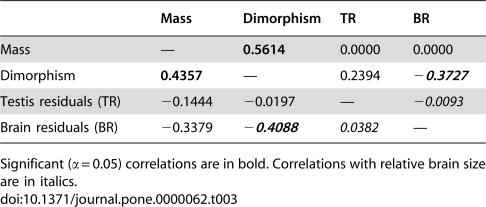
Pearson correlation coefficients among variables (listed above the diagonal) and independent contrasts (listed below the diagonal)

	Mass	Dimorphism	TR	BR
Mass	—	**0.5614**	0.0000	0.0000
Dimorphism	**0.4357**	—	0.2394	**−*0.3727***
Testis residuals (TR)	−0.1444	−0.0197	—	−*0.0093*
Brain residuals (BR)	−0.3379	**−*0.4088***	*0.0382*	—

Significant (α = 0.05) correlations are in bold. Correlations with relative brain size are in italics.

## Discussion

The results of the study indicate that unlike bats, sperm competition did not significantly influence the evolution of brain size in primates. Male competition for access to fertile females, however, is associated with primate brain size evolution, with increasing levels of mass dimorphism associated with decreasing relative brain size.

Mass dimorphism in primates is reported to be correlated strongly with the intensity of male competition, which is proportional to the ratio of reproductively active males to active females [8]. This ratio, often termed the operational sex ratio, is largely dependent on the mating period for females, which in primates is a function of the number and length of estrus cycles experienced before conception [8]. The intensity of male competition in a population—and by extension the level of mass dimorphism—is therefore in part a function of the average length and number of estrus cycles before conception. To test this hypothesis, a *post hoc* analysis of the relationship between dimorphism and temporal availability of fertile females using least squares regression through the origin was conducted. The results of these *post hoc* tests indicated that female availability among primates species does not covary significantly with the average number of days in estrus annually (*b* = 0.039, *r* = 0.023, *p* = 0.965), or with the inter-birth interval (*b* = 0.026, *r* = 0.038, *p* = 0.833) after the phylogenetic effects are accounted for. The relationship between the contrasts for the average number of days of estrous and relative brain size was also not significant (*b* = 0.234, *r* = 0.181, *p* = 0.583), nor was the relationship between relative brain size and the inter-birth interval (*b* = 0.271, *r* = 0.274, *p* = 0.227). These findings suggest that some other component of sexual mass dimorphism that is independent of female availability is likely influencing the evolution of brain size in primates.

Superficially, these results seem to not support the social complexity model for the evolution of larger brain size in primates [2], because monogamy is associated with larger brain sizes than that observed for presumably more complex mating systems such as multi-male/multi-female or single male/multi-female—even after humans are excluded from the analysis. It is important to note however, the social brain hypothesis [2] predicts a strong positive relationship between social complexity as measured by group size, and neocortex size, rather than total brain size. Although total brain size is a proportional measure of neocortex size in primates, future research should incorporate brain organization and tissue type in the analysis of sexual selection and brain evolution.

In their study on testis size and brain evolution in bats Pitnick et al. [7] explain that the expensive tissue hypothesis predicts more intense sexual selection will constrain the evolution of larger brains as a result of energetic trade-offs with costly sexual organs such as testis. Because the present study indicates that relative testis size is not associated with brain size evolution as in bats, the expensive tissue hypotheses as a plausible explanation for brain size evolution in primates is rejected. This finding is perhaps not surprising given the small volume of testicular tissue relative to brain tissue in anthropoid primates. Similar to the study of brain size evolution in bats [7], the present results do not support the recently proposed sexual conflict hypothesis which states that both males and females are under selection to subvert the reproductive investment made by the other sex. As recently summarized by Pitnick et al. [7], the sexual conflict hypothesis predicts species with promiscuous breeding will have larger relative brain sizes than those who breed monogamously. The present study indicates that for primates, just the opposite is found, monogamy is associated with larger relative brain sizes.

The interpretation that the results of the present study are inconsistent with the social brain hypothesis assumes that multi-male/multi-female mating systems are more complex than monogamous systems. If, however, monogamous mating systems require greater social acuity and abilities for deception and social or psychological manipulation, then monogamy would select for larger and potentially more complex brains. Such selection would be associated with lower levels of male competition and would operate independent of sperm competition. If accurate, these interpretations suggest the present study actually supports a social complexity model for primate evolution, with the caveat that group size may not always be the best indicator of all forms social complexity in primates. Additional data on brain size from monogamous and polyandrous primate species are needed to test further the nuanced relationships between the evolution of brain size, sexual selection and social complexity.

## Methods

For the present study, relative testes size, or the gonadosomatic index, was used as a measure of the level of sperm competition [9]–[12], and sexual mass dimorphism was used as a measure of the level of male competition for mates [13]. Data on body mass, testis weight, brain weight, mating systems, and female promiscuity for 30 species of primates, including humans were gathered from several sources [9], [14] (Table S1). As a result, the data on brain and testes size do not originate from the same subjects. Data were not available for all variables for all taxa. Testes weights represent the combined mass of both testes. Species averages for brain weight comprise both males and females. Body mass dimorphism was calculated as the ratio of the male to female body mass. Relative brain and testis sizes were represented by the residuals of brain and testis size regressed on male body mass using the least-squares model. Female promiscuity and the form of mating system for a given taxon was determined from accounts in the literature [14] and coded as categorical variables. *Post hoc*, or secondary analyses, were conducted on published data on estrus cycle length and inter-birth interval (Table S2) after initial findings on the relationship between sexual dimorphism and relative brain size.

### Statistical analysis

Like all mammals, brain size and testes size scale allometrically with body size in primates [15]. Much of the variation in brain and testes sizes among primate taxa, therefore, is attributable to selection for body size. An analysis of covariance (ANCOVA) model with body mass as a covariate was used to assess the effect of mating system and female promiscuity on brain size and testes size in the sample after accounting for body mass. These effects were illustrated graphically using the residuals from the linear regression models of brain and testis weights on male body mass. The effect of body size dimorphism was examined using multiple regression with brain and testis size as the dependent variables and male body size and mass dimorphism as independent covariables. Bootstrapping was used to generate bias-free estimates of regression and correlation coefficients. All variables were log_e_ transformed prior to analysis, and an arbitrary two-tailed significance level of α. = 0.05 was used for all tests.

Before employing a phylogenetically based comparative method, phylogenetic autocorrelation in relative brain size, body mass, and relative testicular size was tested using Phylogenetic Independence version 2.0 [16], [17]. The results of these tests (not shown) indicated these variables were significantly correlated with evolutionary history. The effects of shared evolutionary history on the relationships among measures of relative brain size, relative testes size, and dimorphism were therefore assessed using phylogenetically independent contrasts [18]. Independent contrasts were estimated using the primate phylogeny presented in Purvis [19] with all branch lengths set to 1 (Text S1). The evolutionary relationship among variables was assessed using Pearson correlation coefficients and least-squares regression through the origin. Analyses of independent contrasts and test diagnostics [20] were conducted with the PDAP:PDTREE module of Mesquite 1.06 [21].

## Supporting Information

Table S1 Primary morphometric data and mating system designations
(0.03 MB DOC)

Table S2 Data on oestrus length and inter-birth intervals
(0.03 MB DOC)

Text S1 Description of phylogenetic tree topology
(0.03 MB DOC)
