# Supplementary material for: Sexual Selection and the Evolution of Brain Size in Primates
Source: PLoS One. 2006 Dec 20;1(1):e62. doi: 10.1371/journal.pone.0000062 (PMC1762360; doi:10.1371/journal.pone.0000062)
Supplement: Table S1 — Primary morphometric data and mating system designations (0.03 MB DOC) [file pone.0000062.s001.doc]

**Table S1.** Primary morphometric data and mating system designations.

All data were taken from Harvey et al., 1987

**Body weight^1^**

**Taxon M/F MD^2^ Testis^3^ Brain^4^ Mating system^5^ FMM^6^**

*Callathrix jacchus* 0.31/0.29 1.069 1.3 7.9 PA Yes

*Saguinus oedipus* 0.49/0.51 0.961 3.4 9.0 PA Yes

*Saimiri sciureus* 0.75/0.58 1.293 3.2 24.4 MMMF Yes

*Aotus trivergatus* 0.92/1 0.920 1.2 18.2 Mon No

*Lagothrix* *lagothricha* 6.8/5.8 1.172 11.2 96.4 MMMF Yes

*Ateles geoffroyi* 6.2/5.8 1.069 13.4 110.9 MMMF Yes

*Aloutta palliata* 7.4/5.7 1.298 23 55.1 MMMF Yes

*Presbytis rubicunda* 6.3/6.3 1.000 3.4 92.7 SM No

*Presbytis cristata* 8.6/8.1 1.062 6.2 64 SM No

*Presbytis obscura* 8.3/6.5 1.277 4.8 67.6 SM No

*Presbytis entellus* 18.4/11.4 1.614 11.1 135.2 SM No

*Nasalis lavartus* 20.3/9.9 2.051 11.8 94.2 SM No

*Colobus polykomos* 10.4/8.4 1.238 10.7 76.7 SM No

*Cercopithecus aethiops* 4.75/3.6 1.319 13 59.8 MMMF Yes

*Macaca fascicularis* 5.9/4.1 1.439 35.2 69.2 MMMF Yes

*Macaca mulatta* 6.2/3 2.067 46.2 95.1 MMMF Yes

*Macaca arctoides* 9.2/8 1.784 48.15 76.8 MMMF Yes

*Macaca radiata* 6.6/3.7 1.150 48.2 104.1 MMMF Yes

*Macaca* *nemestrina* 10.4/7.8 1.333 66.7 106 MMMF Yes

*Papio anubis* 21/12 1.750 93.5 175.1 MMMF Yes

*Papio papio* 26/13 2.000 88.9 165.3 MMMF Yes

*Papio cynocephalus* 20/15 1.333 52 169.1 MMMF Yes

*Papio ursinus* 20.4/16.8 1.214 72 214.4 MMMF Yes

*Papio hamadryas* 21.5/9.4 2.287 27.1 142.5 SM No

*Theropithecus gelada* 20.5/13.6 1.507 17.1 131.9 SM No

*Hylobates moloch* 6/5.7 1.053 6.1 113.7 Mon No

*Hylobates lar* 5.7/5.3 1.075 5.5 107.7 Mon No

*Pongo pygmaeus* 69/37 1.865 35.3 413.3 SM No

*Gorilla gorilla* 160/93 1.720 29.6 505.9 SM No

*Pan troglodytes* 41.6/31.1 1.338 118.8 410.3 MMMF Yes

*Homo sapiens* 47.9/40.1 1.195 40.5 1250 Mon No

^1^ male (M) and female (F) body weight in kilograms

^2^ Mass dimorphism (male mass/female mass)

^3^ Testis weight (grams)

^4^ Brain weight (grams)

^5^ Mating system: MMMF, multi-male/multi-female; PA, polyandrous; Mon, monogamous; SM single male.

^6^ presence of multiple female matings as the norm (female promiscuity).
