# Supplementary material for: Sexual Selection and the Evolution of Brain Size in Primates
Source: PLoS One. 2006 Dec 20;1(1):e62. doi: 10.1371/journal.pone.0000062 (PMC1762360; doi:10.1371/journal.pone.0000062)
Supplement: Table S2 — Data on oestrus length and inter-birth intervals (0.03 MB DOC) [file pone.0000062.s002.doc]

**Table S2.** Data on oestrus length and inter-birth intervals.

All data were taken from Harvey et al., 1987

**Taxon Oestrus cycle length (days) Inter-birth interval (days)**

*Callathrix jacchus* 16 157

*Saguinus oedipus*  16 280

*Saimiri sciureus*  18 414

*Aotus trivergatus*

*Lagothrix* *lagothricha* 25 720

*Ateles geoffroyi*  26 870

*Aloutta palliata* 16 675

*Presbytis rubicunda*

*Presbytis cristata*

*Presbytis obscura*

*Presbytis entellus*  22

*Nasalis lavartus*

*Colobus polykomos*

*Cercopithecus aethiops*  33 365

*Macaca fascicularis*  28 390

*Macaca mulatta*  29 360

*Macaca arctoides*  29 525

*Macaca radiata* 28

*Macaca* *nemestrina*  28 405

*Papio anubis*  31 420

*Papio papio*  423

*Papio cynocephalus*  31 630

*Papio ursinus*

*Papio hamadryas*

*Theropithecus gelada*  34 525

*Hylobates moloch*

*Hyobates lar*  27 969

*Pongo pygmaeus*  30 1025

*Gorilla gorilla* 28 1460

*Pan troglodytes* 36 1825

*Homo sapiens*  28 1440
