# Supplementary material for: Sexual Selection and the Evolution of Brain Size in Primates
Source: PLoS One. 2006 Dec 20;1(1):e62. doi: 10.1371/journal.pone.0000062 (PMC1762360; doi:10.1371/journal.pone.0000062)
Supplement: Text S1 — Description of phylogenetic tree topology (0.03 MB DOC) [file pone.0000062.s003.doc]

**Text S1:** Description of phylogenetic tree topology

(((((*Callathrix jacchus*, *Saguinus oedipus*) *Saimiri sciureus* ) *Aotus trivergatus*)((*Lagothrix* *lagothricha*, *Ateles geoffroyi*) *Aloutta palliata*))((((((*Presbytis rubicunda*, *Presbytis cristata*, *Presbytis obscura*) *Presbytis entellus*) *Nasalis lavartus*), *Colobus polykomos*) (((((*Macaca mulatta*, *Macaca fascicularis*)(*Macaca arctoides*, *Macaca radiata*))*Macaca* *nemestrina*)((((*Papio anubis*, *Papio papio*) *Papio cynocephalus*, *Papio ursinus*) *Papio hamadryas*) *Theropithecus gelada*)) *Cercopithecus aethiops*))((*Hylobates moloch*, *Hyobates lar*)( *Pongo pygmaeus* (*Gorilla gorilla* (*Pan troglodytes*, *Homo sapiens*))))))
